# Modulation of adult hippocampal neurogenesis by interleukin 1 signaling^☆^

**DOI:** 10.1016/j.nbscr.2025.100123

**Published:** 2025-04-25

**Authors:** Maria I. Smirnova, Ning Quan

**Affiliations:** a Department of Biomedical Science, Charles E. Schmidt College of Medicine, Florida Atlantic University, 5353 Parkside Drive, Jupiter, FL, 33458, USA; b Stiles-Nicholson Brain Institute, Florida Atlantic University, Jupiter, FL, 33458, USA

## Abstract

Adult hippocampal neurogenesis (AHN) plays a critical role in cognition and emotional regulation. Recent studies have linked compromised AHN to numerous neurological and psychological disorders. The actions of the inflammatory cytokine interleukin-1 (IL-1) have been found to suppress AHN and antagonism of IL-1 signaling has been advocated as a therapeutic strategy for the treatment of neurodegenerative diseases and affective disorders. On the other hand, work from Jim Krueger's group revealed the physiological function of IL-1 in brain homeostasis, indicating the potential downside of IL-1 blockade. Current literature also shows AHN participates in normal functions of the brain in parallel to IL-1. This mini-review analyzes how IL-1 might positively or negatively modulate AHN and the implications of the relationship between IL-1 and AHN on health and disease. Specifically, we will highlight the parallels between IL-1 signaling and AHN in physiological and disease states. We propose that IL-1 signaling modulates AHN in a context-dependent manner; whereas its elevated signaling impairs neurogenesis and contributes to neurological and psychiatric disorders, its physiological role suggests potential therapeutic benefits of IL-1 antagonism must consider the preservation of the beneficial actions of IL-1.

## Highlights

• IL-1 and adult hippocampal neurogenesis both affect cognitive behavior.
• Various CNS disease states are associated with changes in brain IL-1 and adult hippocampal neurogenesis.
• IL-1 can positively and negatively influence adult hippocampal neurogenesis depending on context.
• IL-1 antagonism can be a beneficial treatment , but the pro-neurogenic IL-1 effects should be preserved.

## 1 Introduction

Adult hippocampal neurogenesis (AHN) is the process by which new neurons are generated in the adult mammalian brain, within the subgranular zone (SGZ) of the dentate gyrus (DG) in the hippocampus (Ming et al., 2005, 2011; Altman et al., 1965; Miller et al., 2013). This process begins early in embryonic development, peaks before birth, and continues into adulthood (Urbán et al., 2014; Imayoshi et al., 2008; Semënov, 2019). Surprisingly, although AHN only produces a tiny fraction of neurons in the overall adult neuronal population and only produces new mature neurons in the dentate gyrus of the hippocampus, it plays a crucial role in learning (Nilsson et al., 1999; Yau et al., 2015), memory (Park et al., 2023; Berdugo-Vega et al., 2020), and emotional regulation (Kirby et al., 2012; Chan et al., 2017). While AHN persists throughout life, its rate declines gradually with age (Kuhn et al., 1996), resulting in low levels of AHN in old age. The functional significance of the low-level neurogenesis in old age remains to be clarified, although reduced AHN parallels cognitive decline during ageing, highlighting the importance of maintaining healthy levels of AHN in sustaining cognitive efficiency.

In young adulthood, neurogenesis supports cognitive functions like spatial memory, cognitive flexibility, and emotional regulation (Okamoto et al., 2021; Gulino et al., 2023; Snyder et al., 2005). Physical activity, environmental enrichment, and stress are known as the major factors influencing AHN. Exercise and environmental enrichment promote AHN (Vivar et al., 2013, 2023; Bekinschtein et al., 2011; Saheb et al., 2023; Van Praag et al., 1999), while stress impairs it (Du Preez et al., 2021; Snyder et al., 2011). In general, increased AHN is associated with improved cognitive function and emotional regulation, whereas decreased AHN is associated with the opposite effects. In old age and in neurodegenerative diseases such as Alzheimer's disease (AD), AHN is significantly reduced (Lazarov et al., 2010; Jin et al., 2021). Remarkably, specific augmentation of AHN alone can rescue cognitive and affective deficits in old animals and in mouse models of AD (Choi et al., 2018), suggesting reduced AHN is a critical pathogenic mechanism of aging- and neurodegeneration-induced behavioral deficits, and treatment that increases AHN is a promising method to counter their detrimental effects.

A large body of literature has detailed 5 stages of AHN: 1) activation of radial glia-like cells in the subgranular zone of the dentate gyrus, 2) proliferation of intermediate progenitors, 3) generation of neuroblasts, 4) generation of immature neurons, and 5) maturation and integration of newborn neurons into the dentate granule cell network. Each of these stages may be positively or negatively regulated by both intrinsic (intracellular regulatory system inside the AHN neurons) and extrinsic (microenvironment that constitutes the neurogenic niche) mechanisms (Ming et al., 2011; Toda et al., 2019). Understanding how these mechanisms can be manipulated will be foundational for the development of strategies to curtail the loss of AHN or to strengthen AHN.

**Fig. 1:**
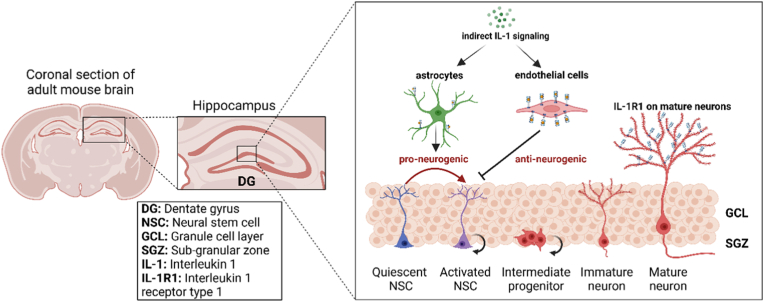
Hypothetical diagram of interleukin 1 (IL-1) signaling on different cell types in the hippocampus influencing adult hippocampal neurogenesis (AHN) at various stages of neural development. Although neural stem cells (NSCs) and immature neurons do not express IL-1 receptor type 1 (IL-1R1), IL-1 signaling can impact the local microenvironment indirectly by acting on surrounding cells, including astrocytes and endothelial cells. These cells can release cytokines, chemokines, or growth factors, which can then alter the hippocampal microenvironment in ways that either enhance or hinder the integration of new neurons. Created in BioRender. https://BioRender.com/h76p022.

The pro-inflammatory cytokine Interleukin-1 (IL-1) is a master regulator of immune responses (Dinarello, 2009). The role of IL-1 in regulating AHN has been investigated as a key neuroimmune modulator, but no systematic analysis has been conducted. IL-1 exists in two forms. Interleukin 1 alpha (IL-1α) is membrane-bound and triggers inflammatory responses to tissue injury (Dinarello, 2018; Cavalli et al., 2021). Interleukin 1 beta (IL-1β) is the secreted form. It has to be cleaved by inflammasomes from the pro-IL-1β to become active and it is released in response to injury, infectious agents, and cellular or systemic stress (Lopez-Castejon et al., 2011). When IL-1 binds to its sole functional receptor (type 1 IL-1 receptor, IL-1R1) on the cell membrane, the IL-1R1/IL-1 complex recruits IL-1 receptor accessory protein (IL1RAcP) and forms an IL-1R1/IL-1RAcP dimer. This dimer activates MyD88 and the downstream IRAK/TRAF pathways that are involved in the immune responses (Martin et al., 2002). Two conventional downstream pathways have been identified: 1) the NFκB pathway, which is involved in the production of cytokines, chemokines, and adhesion molecules, is a major amplifier of inflammatory responses; 2) the mitogen-activated protein kinases (MAPKs) pathway, which is mediated by p38, JNK, and ERK, is known to cause both the expression of pro-inflammatory genes in the immune cells and the modulation of neuronal plasticity in neurons (Weber et al., 2010). IL-1 signaling activates immune cells such as macrophages and T cells, leading to the initiation of both innate and adaptive immunity to sites of infection or injury (Basu et al., 2004). Given its central role in inflammation, IL-1 has become a target for therapeutic interventions. Inhibitors of IL-1, such as anakinra (IL-1 receptor antagonist, IL-1ra) and canakinumab (a monoclonal antibody against IL-1β), are used to treat conditions like rheumatoid arthritis and certain autoinflammatory syndromes (Gaggiano et al., 2021; Sanz-Cabanillas et al., 2023). These treatments have been used to reduce inflammation and alleviate symptoms in conditions where IL-1 is overactive. The consequences of these treatments in regard to AHN have only been explored sporadically. Possible interfaces between IL-1 signaling and AHN are diagrammed in Fig. 1.

## 2 IL-1 signaling affects AHN in disease states

The relationship between IL-1 signaling and neurogenesis was first probed in *in vitro* studies in which cultured neural progenitor cells (NPC) were treated with IL-1α or IL-1β. These studies were expertly reviewed previously and both stimulatory and inhibitory effects of IL-1 on NPC proliferation and differentiation were found (Ryan et al., 2013; Green et al., 2012). It should be noted that these studies were not able to verify whether the cultured NPCs, often derived from primary culture of neonatal animals, truly resemble those in adult hippocampus. Indeed, our recent study using an IL-1R1 reporter mouse showed that immature neurons in the adult SGZ do not express IL-1R1 until they become mature neurons (Liu et al., 2019). Therefore, direct action of IL-1 on NPCs in the AHN is not likely and therefore, this review will primarily focus on the impact of IL-1 signaling on AHN *in vivo*.

The importance of IL-1 signaling on AHN can be derived from two parallel tracks of investigations examining the impact of IL-1 and AHN respectively in various disease states. IL-1 is produced mostly by microglia and macrophages in the brain (Dinarello, 2011; Garlanda et al., 2013). Occasionally, IL-1 was found to be produced by astrocytes and endothelial cells (Becher et al., 2017; McAfoose et al., 2009) during neural injury or infection. In the central nervous system, besides its conventional roles in the immune responses (Dubé et al., 2005) (Rothwell, 2003), IL-1 signaling is involved in pathological conditions such as neurodegeneration (Shaftel et al., 2008), anxiety disorders and major depression (Murray et al., 2013; DiSabato et al., 2021) as well as stress-induced psychopathologies (Goshen et al., 2009). This is not surprising because neuroinflammation is a common pathogenic mechanism for all of the above-mentioned conditions. Specifically, neuroinflammation-associated elevation of IL-1 signaling has been detected in Alzheimer's disease, Parkinson's disease, traumatic brain injury, cerebral ischemia, major depression, anxiety disorders, and PTSD (Shaftel et al., 2008; Griffin et al., 1989; Sokolova et al., 2009; Schädlich et al., 2022; Stojakovic et al., 2017). In parallel, AHN is also significantly altered in these disease conditions (Amanollahi et al., 2023; Wang et al., 2016; Li et al., 2020, 2022; Mahar et al., 2014; Fujikawa et al., 2024). Whether IL-1 signaling contributes to the changes in AHN, which in turn cause behavioral and neurological deficits appears to depend on specific context.

## 3 TBI, AHN, and IL-1

Traumatic brain injury (TBI) is one condition where AHN is disrupted. Brain injuries, especially those involving the hippocampus, can lead to long-term cognitive and emotional regulation deficits, including memory deficits, impaired learning, and mood disorders (Wang et al., 2016, 2025). It is well documented that hippocampal tissue injury causes local inflammation and increased IL-1 expression near the dentate gyrus, the site of AHN. Numerous studies showed in TBI models that AHN is significantly increased following TBI (Bielefeld et al., 2024; Campbell et al., 2024) and the severity of injury correlates with the amounts of increased in AHN (Redell et al., 2020). These findings, however, do not imply that increased ANH is a cause of disease progression, because promoting neurogenesis in the aftermath of TBI was shown to improve recovery and help restore some of the lost cognitive functions (Zhang et al., 2022). Therefore, increased AHN after TBI could represent a reparatory response of the brain that attempts to recover from the loss of network function after the injury. This response, however, does not always result in normal AHN as increased dendritic branching structures of the newborn neurons and their ectopic localization in the hilus region, in addition to the dentate granule cell layer, was observed (McCloskey et al., 2006; Overstreet-Wadiche et al., 2006; LaSarge et al., 2016). One of the negative consequences of the abnormal AHN after TBI is the increased sensitivity to epileptogenesis (Webster et al., 2017). Studies have revealed several potential mechanisms for TBI induced increase in AHN: action of locally produced nerve growth factors such as fibroblast growth factor 2 (FGF2) and brain-derived neurotrophic factor (BDNF), increased neuronal activity such as heightened glutamatergic inputs, and endogenous neuronal loss. On balance, post-TBI neurogenesis does not represent an excessive host response and treatments for further enhancement of AHN appear to be beneficial.

TBI also inevitably causes an increased expression of IL-1. Injured neurons, glial cells and endothelial cells release damage-associated molecular patterns (DAMPs), a set of molecules also called alarmins. DAMPs then directly or indirectly drive the expression of IL-1, primarily from brain microglia or infiltrating leukocytes near the site of injury (Martin, 2016). Downstream of IL-1 signaling, IL-1 drives the expression of adhesion molecules and chemokines from cerebral blood vessels, induces the production of reactive oxygen species (ROS), and contributes to the breakdown of the blood brain barrier (BBB). Previous studies by and large showed that blocking IL-1 signaling via IL-1 receptor antagonist, antibodies against IL-1β, or deletion of IL-1R1 provided neuroprotection and facilitated cognitive recovery after TBI (Tehranian et al., 2002; Helmy et al., 2014).

Whether TBI associated expression of IL-1 is pro- or anti-AHN is unclear. In fact, no study has simultaneously examined AHN and IL-1 signaling in the same TBI model. The association between increased neurogenesis and the expression of IL-1 would suggest that IL-1 could be pro-neurogenic in TBI, but the fact that inhibition of IL-1signaling facilitated cognitive recovery in TBI would suggest the contrary because increased neurogenesis is associated with better cognitive outcomes. Two caveats can be considered: 1) TBI generates a transient increase of IL-1 which can quickly dissipate in days, but post-TBI AHN was typically measured much later, therefore, the early expression of IL-1 could have produced an immediate anti-neurogenic effect, unassociated with the later post-TBI repair mechanisms that promoted AHN when IL-1 expression is no longer increased in the brain; 2) post-TBI IL-1 expression did promote AHN, but anti-IL-1 treatments are likely to affect much broader sets of IL-1-mediated neuroinflammatory events, e.g., reduction of ROS production, diminution of leukocyte infiltration and prevention of BBB damage, such that these non-AHN related neuroprotective effects of IL-1 antagonism outweighed its potential anti-AHN effects. Nevertheless, future studies need to clarify whether IL-1 is pro- or anti-AHN in TBI in order to refine the potential benefit of anti-IL-1 therapy.

## 4 Depression, AHN, and IL -1

Studies in the area of psychological depression presented a different scenario. Classical theory on the pathogenesis of depression posited that the lack of synaptic serotonin in the brain is a main cause of depression (Nutt, 2008). Drugs that inhibit serotonin re-uptake (SSRI) to allow more extracellular serotonin to be available have proven to be clinically effective for the treatment of depression (Preskorn, 1997). Recent studies, however, have added additional dimensions to the understanding of the pathogenic mechanisms of depression. In both human and animal model studies of depression, reduced AHN have been found in depressed subjects. In fact, smaller hippocampal volumes, a potential consequence of reduced AHN, have been associated with patients of depression (Videbech et al., 2004; Barch et al., 2019). In addition, depressed patients often exhibit cognitive deficits (Guo et al., 2024). This makes sense because serotonergic (emotional) and hippocampal (cognitive) circuits are known to reciprocally regulate each other and dysfunctional cognitive control of emotion could be part of neural mechanism for depression. In this vein, hippocampal regulated prefrontal cortex (PFC) shows reduced plasticity in animal models of depression and serotonin stimulation of PFC can reduce depressive behaviors in animal models. Remarkably, treatment with antidepressants has been found to promote AHN and specific pharmacological or genetic enhancement of AHN was found to buffer stress induced depressive behavior (Tunc-Ozcan et al., 2019; Rahmani et al., 2013; Hovorka et al., 2022). Thus, AHN could be an exciting new target for the treatment of depression.

Accumulated new evidence also suggests that neuroinflammation and specifically brain IL-1 signaling could contribute to the development of depression (Wu et al., 2023; Farooq et al., 2017). Many symptoms in depression resemble sickness behaviors after infection such anhedonia, fatigue, and reduced social interaction, all of which can be induced by brain IL-1 signaling (DiSabato et al., 2021; Cady et al., 1989; Roerink et al., 2017). In addition, in some animal models of depression, brain IL-1β expression was increased and treatments with IL-1ra, deletion of IL-1R1, or inhibition of inflammasome activities, required for the production of active IL-1β, have been shown to reduce depressive-like behaviors (Barrientos et al., 2003; Li et al., 2021, 2023; Koo et al., 2009; Xia et al., 2023). These results suggest antagonism of IL-1 signaling is similarly beneficial for the treatment of depression as augmentation of AHN.

Is IL-1 signaling related to the reduced AHN seen in depression? Has IL-1 antagonism that was effective in reducing depressive behaviors resulted from augmented AHN? Very few studies examined depression, AHN and IL-1 signaling together *in vivo*. Two studies, one in rats and another in mice, showed acute or chronic stress causes decreases in AHN, which were associated with anhedonia, one of the depressive-like behaviors (Goshen et al., 2008). Both the reduced AHN and the anhedonia were blocked by IL-1R1 deletion or treatment with IL-1ra. The authors concluded that IL-1 signaling is required for the anti-neurogenic effects seen in depression. Certain caveats need to be considered. For example, acute stress is known to induce the immunosuppressive glucocorticoids that can inhibit proliferation of stem cells (Zhang et al., 2023; Tartt et al., 2022) and the expression of IL-1, raising the possibility that the anti-neurogenic effects in the stress-induced depression models were not a consequence of IL-1, but glucocorticoid, signaling and that brain IL-1 should be decreased, not increased, in these models. However, the authors of these studies showed that stress, and surprisingly even direct administration of glucocorticoids, induced brain IL-1β expression and the glucocorticoid-induced anti-neurogenic effects required brain IL-1 signaling. Indeed, other studies have shown that stress can paradoxically trigger neuroinflammation by first activating the immunosuppressive hypothalamic-pituitary-adrenal (HPA) axis and then activating the proinflammatory brain IL-1 expression, which causes more cortisol release (Goshen et al., 2008), sustaining a perpetual neuroinflammatory cycle. These studies did not measure glucocorticoids and brain IL-1 at the same time, leaving the possibility that the increase of brain IL-1 after stress exposure was due to a later rebound of the system after stress-induced initial decrease of brain IL-1. It should be noted stress-induced HPA activation show habituation over time, such that repetition of the same stress stimulations causes diminished responses (McEwen, 1998; Roos et al., 2019). This could be why chronic variable or unpredictable stress is required to generate animal models of depression such that the elevated brain neuroinflammation might be maintained. Presumably chronically elevated brain IL-1 so ensued can be anti-neurogenic and contributes behavioral depression.

## 5 PTSD, AHN, and IL-1

The link between AHN and the post-traumatic stress disorder (PTSD) might be the most intuitive in terms of behavioral phenotype. PTSD patients can be triggered by painful previous traumatic memory and have difficulty discriminating truly dangerous cues from innocuous background signals that happened to be present at the traumatic event (Thakur et al., 2022). Dentate granule cells are known to play a critical role in pattern separation (Bakker et al., 2008), which could be crucial for the prevention or alleviation of PTSD symptoms by discriminating sensory cues from previous sensory patterns associated with the traumatic events. An elegant recent study by Tuncdemir et al. showed that newborn granule cells promote such pattern separation through the formation of non-overlapping presentations of identical sensory cues at different locations (Tuncdemir et al., 2023). Deletion of AHN resulted in overgeneralization of anxiety. In addition, AHN was found to favor contextual learning over procedural learning, thus allowing the changing external environment to place higher saliency than previously established internal reference (Berdugo-Vega et al., 2020, 2021). Such cognitive flexibility could mitigate PTSD. Studies have shown that individuals with PTSD indeed have lower levels of AHN (Yagi et al., 2022). This reduction, through the above-mentioned mechanisms, may contribute to the difficulty in processing and distinguishing past trauma from present experiences, a hallmark of PTSD. Impaired AHN may also make it harder for individuals to adapt to new emotional information, leading to persistent symptoms of anxiety, hypervigilance, and flashbacks. Ongoing research shows enhancing AHN could serve as a potential treatment for PTSD, with strategies like exercise and stress reduction showing promise in promoting neurogenesis and improving emotional resilience (Chang et al., 2024; Sun et al., 2020, 2021; Ishikawa et al., 2019).

In individuals with PTSD, IL-1 may contribute to the persistent inflammation and altered brain function sometimes observed in this disorder. Interestingly, although one human study in earthquake survivors showed IL-1β levels are correlated with PTSD severity (Wang et al., 2019), other studies have not consistently found increased baseline IL-1 level in PTSD (Küffer et al., 2019). PTSD can manifest after traumatic events that occurred in distant past, sometimes even in childhood, potentially allowing baseline IL-1 to recover from the impact of the trauma from long ago. However, in both human and animal models of PTSD, a more consistent finding is that IL-1 levels are exaggerated after a later hit of stress or infection, suggesting previous traumatic event may have caused epigenetic changes that reprogram the system to exhibit heightened IL-1 expression upon later stress or immune challenges (Uddin et al., 2010). The resultant hyper-inflammatory activity is then correlated with dysregulated stress responses, impaired recovery and the development of PTSD symptoms, such as hyperarousal, intrusive thoughts, and avoidance behavior (Jones et al., 2018). The causal relationship between IL-1 and PTSD was suggested by one mechanistic study, which found that in the stress-enhanced fear learning model of PTSD, IL-1 signaling is required for the increased fear response which was reduced by IL-1ra or by morphine treatment that reduced IL-1β expression in the hippocampus (Jones et al., 2018; Szczytkowski-Thomson et al., 2013).

Whether IL-1 signaling mediates PTSD-related reduction in AHN is unclear. Most studies only investigated the consequence of acute or chronic stress on AHN immediately after the stress exposure to infer the mechanisms for PTSD. It should be noted that PTSD could occur long after the initial stressful event. In addition, not all acute stresses resulted in reduced AHN, depending on the type of acute stress that was applied, and AHN can recover days after the acute stress exposure (Aguayo et al., 2018; Kirby et al., 2013). Chronic mild stress and unpredictable stress have been shown to cause a reduction in AHN; but how long will this effect last is unclear. Typically, stress-induced anti-neurogenic effects require glucocorticoids and immature neurons express very low levels of glucocorticoid receptor (Saaltink et al., 2014). Therefore, glucocorticoid might exert its anti-neurogenic effects indirectly. Two studies suggested that IL-1 signaling, downstream of glucocorticoids, mediates stress induced suppression of AHN (Goshen et al., 2008; Koo et al., 2008). Because IL-1R1 is not expressed in NPCs and immature neurons of the dentate gyrus, such effect may also be mediated indirectly via IL-1R1 expressed on the non-neuronal cells in the hippocampus.

It is possible combining AHN augmentation, e.g., stimulating BDNF signaling, with IL-1 antagonism that reduces hyper-IL-1-signaling induced suppression of AHN could be an effective therapeutic strategy for the treatment of PTSD.

## 6 Alzheimer's disease, AHN, and IL-1

Loss of AHN is implicated in neurodegenerative diseases, such as Alzheimer's disease (AD) (Sung et al., 2020; Vivar, 2015). Although earlier studies from human pathology have emphasized the loss of cortical cholinergic neurons in AD, the hippocampus, a key brain region involved in memory formation, is also particularly affected in AD. Hallmark symptoms of AD including memory loss and cognitive impairment may arise specifically from neural circuit damages of the hippocampus (Zhang et al., 2024). The accumulation of amyloid-beta plaques and tau tangles—two hallmarks of Alzheimer's pathology—has been shown to disrupt the environment necessary for the proper functioning of neural stem cells and progenitor cells in the subgranular zone (SGZ), hindering neurogenesis (Moreno-Jiménez et al., 2019; Abbate, 2023). This disruption in AHN is now believed to exacerbate the cognitive decline in AD. Several studies showed that promoting AHN can help counteract neurodegenerative processes and improve cognitive function (Choi et al., 2018; Singh et al., 2012; Mishra et al., 2022). In fact, interventions aimed at boosting AHN (such as physical exercise or certain pharmaceutical compounds) have been explored as potential therapies to delay the onset of AD or mitigate its symptoms (Choi et al., 2018; Singh et al., 2012; Mishra et al., 2022; Walgrave et al., 2021).

In AD, IL-1β is often elevated and contributes to the neuroinflammatory processes that were thought to accelerate amyloid plaque accumulation and tau pathology (Shaftel et al., 2008; Griffin et al., 1989, 2006). Systemic immune challenge in AD subjects promotes neuronal damage and cognitive decline via IL-1β-induced activation of microglia and astrocytes (Lopez-Rodriguez et al., 2021). Blocking IL-1 signaling has been shown to rescue cognition and attenuate Tau pathology in an animal model of AD (Kitazawa et al., 2011), strongly suggesting that IL-1 contributes to the pathogenesis of AD. In accordance, animals with IL-1ra deletion show exaggerated AD progression. Further, chronic IL-1 activation leads to sustained neuroinflammation, impairing neuroplasticity and contributing to cognitive decline (Griffin et al., 1989; Dinarello et al., 1993). On the other hand, chronic overexpression of IL-1β in animal models of AD resulted in the clearance of amyloid plaques due to activation of microglia that are important for phagocytosing misfolded proteins in the brain (Shaftel et al., 2007), demonstrating the neuroprotective effects of IL-1 in AD. Thus, simply blocking IL-1 signaling may not be advisable for the treatment of AD because both strong detrimental and beneficial effects have been demonstrated for this cytokine in AD.

How does IL-1 signaling affect AHN in AD? A series of studies from Yirmiya's group addressed this issue with intra-hippocampal transplantation of NPCs. Transplanting wild type NPCs increased AHN in a mouse model of AD and transplanting NPCs with IL-1ra-expressing transgene further elevated AHN, increased BDNF expression and significantly improved cognitive function in the AD mice (Ben-Menachem-Zidon et al., 2014). In addition, this procedure also reduced local plaque burden. It is unclear why IL-1ra treatment reduced plaque burden in this study, contrary to the observation that IL-1 promotes microglia-mediated plaque clearance. Nonetheless, the authors suggested that antagonizing IL-1 signaling in the hippocampus promoted AHN in AD. It should be noted that transplanting WT NPCs also exerted similar, albeit smaller, effects. Therefore, synergistic effects of AHN augmentation and hippocampal IL-1 antagonism, rather than manipulating AHN and IL-1 signaling separately, appear to be more beneficial in AD.

The seemingly contradictory effects of IL-1 signaling in AD could be reconciled by the specific sites of IL-1 signaling. Our un-published observation using an IL-1β reporter mouse we recently created show physiological IL-1β is expressed in the choroid plexus whereas plaque associated IL-1β expression in an AD model is expressed by microglia surrounding the plaques. Whether physiological IL-1β acts on cells in the choroid plexus to remove pathogenic Aβ whereas plaque associated IL-1β acts at the sites of plaque formation on nearby cells to exacerbate plaque pathology remains to be determined.

Overall, disease related IL-1 expression in the hippocampus may be associated with either an increase or a decrease of AHN, depending on the context of specific disease models. The majority of the literature suggest IL-1 antagonism is beneficial especially when overt neuroinflammation is present in the brain. However, under conditions where IL-1 signaling promotes AHN as a physiological function or as a repair mechanism, a deeper understanding of the role of IL-1 in modulating AHN is required to avoid the potential side effects of IL-1 blockade.

## 7 Regulation of the physiological processes of AHN by IL-1

Does IL-1 regulate AHN under physiological conditions? This question has been addressed only indirectly from several lines of research. First, although many early studies failed to detect IL-1 expression in the normal brain (Quan et al., 1996, 1998), minute amounts of biologically active IL-1 have been detected with more sensitive assays (Quan et al., 1999) and with more advance molecular methods such as single cell sequencing (Dani et al., 2021). Although it is difficult to directly study the effects of low levels of brain IL-1, overexpression of IL-1ra in the normal brain to antagonize the endogenous low levels of IL-1 was found to reduce AHN, suggesting physiological IL-1 promotes AHN (Spulber et al., 2008). Second, IL-1 has been directly administered into the brain to test its effects on AHN. One study suggests that at a high dose of IL-1, NSCs take on a gliogenic fate, instead of a neurogenic fate (Wu et al., 2012). This dose is most likely lower than the dose of IL-1 that ablates AHN completely (Soung et al., 2022), probably representing a hypra-physiological level of IL-1. There are no studies that directly show IL-1 promotes AHN; however, at low levels, IL-1 exerts beneficial effects on cognition and plays a role in learning and memory in the healthy brain (Koo et al., 2008; Goshen et al., 2007; Schneider et al., 1998; Yirmiya et al., 2002). Because AHN contributes to learning and memory (Yau et al., 2015), these results again suggest that at low levels, IL-1 promotes AHN.

## 8 Studies from Krueger's group revealed the role of homeostatic IL-1 in physiological sleep

The third line of research that provided inference for the pro-neurogenic effects of homeostatic IL-1 is a body of work from Krueger's group that revealed the role of IL-1 on circadian rhythms and sleep. Brain IL-1 was found to regulate physiological sleep and its expression is also regulated by circadian rhythms, such that higher cerebrospinal fluid IL-1 levels were found during sleep (Krueger et al., 1998). In addition, administration of physiological levels of IL-1 into the brain promoted slow wake sleep (Opp et al., 1991). Furthermore, they demonstrated that the neuronal specific IL-1 receptor accessory protein (IL-1RAcpb) is required for homeostatic sleep (Davis et al., 2015). In parallel, AHN is also regulated by circadian rhythm; AHN shows peak activity at certain times of the day in mice, typically during the light phase (when animals are inactive or sleeping) and decreases during the dark phase. Thus, higher levels of brain IL-1 during sleep correlate with increased AHN during this circadian time. Both AHN and IL-1 have been implicated in promoting normal cognitive functions. For example, the learning process itself induces hippocampal IL-1 expression (Depino et al., 2004) and AHN was found to be required for dentate-dependent pattern separation. Taken together, these findings suggest physiological brain IL-1 promotes AHN in a circadian rhythm-dependent manner, which is required for optimal cognitive function.

## 9 Choroid plexus, astrocytic IL-1R1, and GABA, a potential mechanism of AHN regulation by homeostatic IL-1

Although IL-1 is generally undetectable in the normal brain, recent single cell sequencing analysis revealed that cells of the choroid plexus express significant IL-1 in the normal brain. Our unpublished observation using a sensitive IL-1β reporter mouse also confirmed that IL-1β is prominently expressed in the choroid plexus while brain parenchymal IL-1β-expressing cells are absent. In addition, we reported previously that ependymal cells of the choroid plexus express IL-1R1 (Liu et al., 2019). Interestingly, choroid plexus is also a region that was identified to regulate circadian rhythm (Quintela et al., 2013). A recent study shows that the choroid plexus can modulate the circadian clock of the suprachiasmatic nucleus (SCN) through the circulation of cerebrospinal fluid (Myung et al., 2018). Disruptions in this circadian regulation can alter hippocampal NSC activation from quiescence to re-entry to the cell cycle (Gengatharan et al., 2021). Whether IL-1 signaling in the choroid plexus promotes AHN remains to be determined. Although this is currently hypothetical, it can be tested by conditional knockout of IL-1β expressing cells in the choroid plexus.

Outside ependymal IL-1R1, our recent studies found IL-1R1 is expressed in astrocytes, endothelial cells, and mature glutamatergic granule cells in the hippocampus, but not in microglia and immature neurons (Liu et al., 2019). Further, over-expressing of IL-1β in the hippocampus via adenoviral expression of IL-1 in animals with restricted IL-1R1 expression on brain endothelial cells resulted in activation of microglial cells, leukocyte infiltration, and suppression of AHN, suggesting endothelial IL-1R1, mediates neuroinflammatory, not the homeostatic pro-neurogenic effects, of IL-1. These findings suggest IL-1 is likely to influence physiological AHN via indirect actions via astrocytic IL-1R1 or via glutamatergic IL-1R1.

A hypothetical mechanism for IL-1 to influence AHN is via GABAergic signaling. The SGZ contains a pool of quiescent neural stem cells (NSCs), known as type 1 radial glia-like cells, that have the potential to enter the cell cycle and divide to produce more NSCs that can mature into neurons over time (Reynolds et al., 1992). GABAergic signaling plays a crucial role in the regulation of neural stem cells (NSCs) and their re-entry into the cell cycle (Overstreet Wadiche et al., 2005; Tozuka et al., 2005). GABA, through activation of GABA-A receptors, modulates the activity of NSCs and their progenitors. Upon activation of GABA-A receptors, GABAergic signaling induces a depolarizing effect (due to chloride influx), which can activate Mitogen-Activated Protein Kinase (MAPK) pathway, driving NSCs into the cell cycle via the expression of cyclin and cyclin-dependent kinases (CDKs) (Dieni et al., 2012; Pellarin et al., 2025). As the neuroblasts mature, GABAergic signaling becomes inhibitory, stabilizing the NSCs and regulating their differentiation. This ensures that NSCs maintain a balanced rate of proliferation and differentiation. IL-1 was found *in vitro* to have a bidirectional effect on GABAergic neurons, either enhancing or inhibiting their activity. This dual regulation of GABA receptor function by IL-1 suggests that its role in modulating GABAergic signaling is complex and context-dependent, potentially depending on factors such as the specific brain region, neuronal activity, or the presence of other signaling molecules (Yan et al., 2015; Bajo et al., 2015). It should be noted that our recent mapping study show IL-1R1 is not expressed on adult GABAergic neurons (Nemeth et al., 2024). Therefore, IL-1 is not likely to activate AHN directly via GABAergic neurons in the hippocampus. Alternatively, IL-1 was found to act on astrocytes to trigger the release of GABA (Shim et al., 2019). This could be a fascinating mechanism by which IL-1 promotes physiological AHN. This hypothesis is currently being tested in our lab.

## 10 Caution against IL-1 antagonism as a therapy

As mentioned above, blockade of IL-1 signaling has been proposed as a therapy for several CNS disorders (Koo et al., 2009; Wang et al., 2011). Considering the importance of the physiological role of brain IL-1, there are certain risks to consider. IL-1 plays a vital role in the body's immune response and in maintaining brain homeostasis (Dinarello, 2009). Complete inhibition of IL-1 signaling could impair the body's ability to respond to infections and tissue injury, potentially increasing susceptibility to infections or hindering the brain's natural repair processes after injury. Additionally, chronic IL-1 blockade could disrupt the neural functions of IL-1 in the brain, potentially leading to unintended consequences, which may exacerbate neurodegenerative diseases. One study has even shown that blockade of IL-1 signaling impairs spatial memory in rats (Yirmiya et al., 2002). These risks highlight the need for more targeted and refined approaches to modulate IL-1 signaling, such as selective inhibition in specific IL-1 activated cell types or at particular time points of a given disease, to preserve its beneficial effects while minimizing harmful outcomes.

## 11 Conclusion

In conclusion, adult hippocampal neurogenesis (AHN) is a vital process that underpins cognitive functions such as learning, memory, and emotional regulation. While neurogenesis persists into adulthood, its rate declines with age and can be disrupted in conditions like Alzheimer's disease, depression, and traumatic brain injury. Understanding the regulation of AHN is key to developing effective therapeutic strategies. In general, disease related IL-1 expression can have anti-neurogenic effects and blocking IL-1 signaling could be a promising therapeutic strategy. On the other hand, physiological IL-1 has pro-neurogenic effects that may be mediated by specific cell-type IL-1R1. Understanding the full spectrum of IL-1's influence on AHN will aid the rational design of IL-1 antagonism that avoids blocking the beneficial pro-neurogenic effects of IL-1. These complex relationships between IL-1 and AHN is summarized in Table 1.

**Table 1:** Parallels between IL-1 and AHN in various brain diseases.

Condition	Positive effects of IL-1	Negative effects of IL-1	Effect of IL-1 on adult hippocampal neurogenesis (AHN)
**Traumatic brain injury (TBI)**	- Initial compensatory and reparative responses to injury (Samatra et al., 2018)	- Destruction of blood brain barrier - Reactive oxygen species - Overt inflammation (Tehranian et al., 2002; Helmy et al., 2014)	**Unclear** - No studies with IL-1 levels and AHN together - Prolonged IL-1 signaling via DAMP activation possibly inhibits AHN (Martin, 2016)
**Depression**	- At low levels, IL-1 signaling improves emotional resilience (Liu et al., 2024)	- Chronic inflammation impairs neural progenitor development (Jeon et al., 2018) - Cognitive and emotional dysfunction may contribute to mood disorder and hippocampal atrophy (Femenía et al., 2012)	**Inhibitory** - Chronic IL-1 signaling, possibly through glucocorticoid activation can impair AHN, contributing to cognitive deficits and emotional dysregulation (Koo et al., 2008)
**Post-traumatic stress disorder (PTSD)**	- Low-dose IL-1 could induce initial immune response that aids recovery from trauma (Lilic et al., 1990)	- Chronic inflammatory state - Contributes to impaired stress regulation and memory (Spivak et al., 1997; Waheed et al., 2018)	**Unclear** - No studies with IL-1 levels and AHN together - IL-1 may impair neurogenesis through prolonged inflammation and contribute to long-term emotional and cognitive dysfunction (Goshen et al., 2008; Lee et al., 2022) - IL-1 can aid in trauma recovery after the initial stressful trigger after the high levels of IL-1 have lowered (Lilic et al., 1990)
**Alzheimer's disease (AD)**	- Modulates immune responses to clear amyloid plaques (Shaftel et al., 2007)	- Chronic inflammation accelerating neuronal degeneration (Adamu et al., 2024) - Activation of reactive microglia and astrocytes leading to neuronal damage (Liddelow et al., 2017) - Impairment of cognitive function Shaftel et al. (2008)	**Mostly Inhibitory** - IL-1 promotes neuroinflammation, which may hinder AHN and exacerbate cognitive decline (Koo et al., 2008; Ekdahl et al., 2003) - Understudied low-level IL-1 signaling in promoting AHN in AD

## 12 Future directions

The complex relationship between IL-1 signaling and adult hippocampal neurogenesis (AHN) presents several important avenues for future research. Understanding how IL-1 can be modulated therapeutically without disrupting its necessary physiological roles is critical for developing targeted treatments for neurological and psychiatric disorders. One critical area of exploration is how cell-type-specific IL-1 signaling can be dissected *in vivo*. The conditional IL-1R1 deletion/expression system offers a powerful method for selectively manipulating IL-1 signaling in specific cell populations, such as neural progenitors, astrocytes, or neurons. This can help clarify IL-1's role in neurogenesis and inflammation at a cellular level. Additionally, *in vivo* imaging techniques such as two-photon imaging can be employed to track the behavior of specific cell types in response to IL-1 signaling. By combining these imaging approaches with fluorescent reporters specific to IL-1 receptor activation, we can observe real-time IL-1 signaling dynamics in live cells. Another key future area of research involves the timing of IL-1 modulation of AHN. The timing of IL-1 activation after injury or disease may be crucial in determining whether its effects on AHN are beneficial or detrimental. Temporal manipulation of IL-1 signaling through optogenetic or chemical-genetic tools can help to investigate how IL-1 impacts neurogenesis at various stages of CNS injury and recovery. Distinguishing between chronic vs. acute IL-1 signaling is another important area of investigation. Determining how short-term versus long-term activation of IL-1 affects AHN and cognitive outcomes could provide insights into therapeutic strategies. Temporal control of IL-1 signaling could clarify whether short bursts of IL-1 might promote beneficial plasticity, while chronic inflammation disrupts neurogenesis and cognition. This distinction is vital for understanding the role of IL-1 in both normal and pathological brain function. Furthermore, there is a need to explore whether selective IL-1 receptor blockade could restore AHN without disrupting the receptor's protective role in other cell types. While IL-1 antagonism has been proposed as a therapeutic approach, selective targeting of IL-1 signaling in specific cell types while preserving its beneficial effects in other contexts is crucial. Developing small molecules or monoclonal antibodies that specifically block harmful IL-1 signaling in certain cell populations could offer a more nuanced therapeutic approach. Finally, understanding the long-term effects of IL-1 modulation on cognitive function and behavior is critical. Chronic modulation of IL-1 may influence brain homeostasis, cognitive function, mood, and behavior. Long-term studies in animal models of neurodegenerative or affective disorders will be important for assessing how altering IL-1 signaling impacts mental health and brain function over time. Behavioral assays in combination with assessments of adult hippocampal neurogenesis will provide valuable insights into the long-term consequences of IL-1 modulation. By addressing these key questions, future research will deepen our understanding of the complex role of IL-1 in adult hippocampal neurogenesis and its broader implications for health and disease. This will ultimately inform the development of targeted therapeutic interventions for neurodegenerative diseases, psychiatric disorders, and brain injuries.

## CRediT authorship contribution statement

**Maria I. Smirnova:** Writing – review & editing, Writing – original draft, Visualization, Conceptualization. **Ning Quan:** Writing – review & editing, Writing – original draft, Conceptualization.

## Declaration of competing interest

The authors declare no conflict of interest.

## Data Availability

No data was used for the research described in the article.

## References

[bib1] Abbate C. (2023). The adult neurogenesis theory of Alzheimer's disease. J Alzheimers Dis.

[bib2] Adamu A. (2024). The role of neuroinflammation in neurodegenerative diseases: current understanding and future therapeutic targets. Front. Aging Neurosci..

[bib3] Aguayo F.I. (2018). Hippocampal memory recovery after acute stress: a behavioral, morphological and molecular study. Front. Mol. Neurosci..

[bib4] Altman J., Das G.D. (1965). Autoradiographic and histological evidence of postnatal hippocampal neurogenesis in rats. J. Comp. Neurol..

[bib5] Amanollahi M. (2023). The dialogue between neuroinflammation and adult neurogenesis: mechanisms involved and alterations in neurological diseases. Mol. Neurobiol..

[bib6] Bajo M. (2015). IL-1 interacts with ethanol effects on GABAergic transmission in the mouse central amygdala. Front. Pharmacol..

[bib7] Bakker A. (2008). Pattern separation in the human hippocampal CA3 and dentate gyrus. Science.

[bib8] Barch D.M. (2019). Hippocampal volume and depression among young children. Psychiatry Res. Neuroimaging..

[bib9] Barrientos R.M. (2003). Brain-derived neurotrophic factor mRNA downregulation produced by social isolation is blocked by intrahippocampal interleukin-1 receptor antagonist. Neuroscience.

[bib10] Basu A., Krady J.K., Levison S.W. (2004). Interleukin-1: a master regulator of neuroinflammation. J. Neurosci. Res..

[bib11] Becher B., Spath S., Goverman J. (2017). Cytokine networks in neuroinflammation. Nat. Rev. Immunol..

[bib12] Bekinschtein P. (2011). Effects of environmental enrichment and voluntary exercise on neurogenesis, learning and memory, and pattern separation: BDNF as a critical variable?. Semin. Cell Dev. Biol..

[bib13] Ben-Menachem-Zidon O. (2014). Intra-hippocampal transplantation of neural precursor cells with transgenic over-expression of IL-1 receptor antagonist rescues memory and neurogenesis impairments in an Alzheimer's disease model. Neuropsychopharmacology.

[bib14] Berdugo-Vega G. (2020). Increasing neurogenesis refines hippocampal activity rejuvenating navigational learning strategies and contextual memory throughout life. Nat. Commun..

[bib15] Berdugo-Vega G. (2021). Adult-born neurons promote cognitive flexibility by improving memory precision and indexing. Hippocampus.

[bib16] Bielefeld P. (2024). Traumatic brain injury promotes neurogenesis at the cost of astrogliogenesis in the adult hippocampus of male mice. Nat. Commun..

[bib17] Cady A.B. (1989). Interleukin-1-induced sleep and febrile responses differentially altered by a muramyl dipeptide derivative. Int. J. Immunopharm..

[bib18] Campbell A. (2024). Enhancing neurogenesis after traumatic brain injury: the role of adenosine kinase inhibition in promoting neuronal survival and differentiation. Exp. Neurol..

[bib19] Cavalli G. (2021). Interleukin 1α: a comprehensive review on the role of IL-1α in the pathogenesis and treatment of autoimmune and inflammatory diseases. Autoimmun. Rev..

[bib20] Chan J.N. (2017). Interaction effect of social isolation and high dose corticosteroid on neurogenesis and emotional behavior. Front. Behav. Neurosci..

[bib21] Chang W.L. (2024). Pharmacological enhancement of adult hippocampal neurogenesis improves behavioral pattern separation in young and aged mice. bioRxiv.

[bib22] Choi S.H. (2018). Combined adult neurogenesis and BDNF mimic exercise effects on cognition in an Alzheimer's mouse model. Science.

[bib23] Dani N. (2021). A cellular and spatial map of the choroid plexus across brain ventricles and ages. Cell.

[bib24] Davis C.J. (2015). The neuron-specific interleukin-1 receptor accessory protein is required for homeostatic sleep and sleep responses to influenza viral challenge in mice. Brain Behav. Immun..

[bib25] Depino A.M. (2004). Learning modulation by endogenous hippocampal IL-1: blockade of endogenous IL-1 facilitates memory formation. Hippocampus.

[bib26] Dieni C.V., Chancey J.H., Overstreet-Wadiche L.S. (2012). Dynamic functions of GABA signaling during granule cell maturation. Front. Neural Circ..

[bib27] Dinarello C.A. (2009). Immunological and inflammatory functions of the interleukin-1 family. Annu. Rev. Immunol..

[bib28] Dinarello C.A. (2011). Interleukin-1 in the pathogenesis and treatment of inflammatory diseases. Blood.

[bib29] Dinarello C.A. (2018). Overview of the IL-1 family in innate inflammation and acquired immunity. Immunol. Rev..

[bib30] Dinarello C.A., Wolff S.M. (1993). The role of interleukin-1 in disease. N. Engl. J. Med..

[bib31] DiSabato D.J. (2021). Interleukin-1 receptor on hippocampal neurons drives social withdrawal and cognitive deficits after chronic social stress. Mol. Psychiatr..

[bib32] Du Preez A. (2021). Chronic stress followed by social isolation promotes depressive-like behaviour, alters microglial and astrocyte biology and reduces hippocampal neurogenesis in male mice. Brain Behav. Immun..

[bib33] Dubé C. (2005). Interleukin-1β contributes to the generation of experimental febrile seizures. Ann. Neurol..

[bib34] Ekdahl C.T. (2003). Inflammation is detrimental for neurogenesis in adult brain. Proc. Natl. Acad. Sci..

[bib35] Farooq R.K. (2017). Role of inflammatory cytokines in depression: focus on interleukin-1β. Biomed Rep.

[bib36] Femenía T. (2012). Dysfunctional hippocampal activity affects emotion and cognition in mood disorders. Brain Res..

[bib37] Fujikawa R. (2024). Neurogenesis-dependent remodeling of hippocampal circuits reduces PTSD-like behaviors in adult mice. Mol. Psychiatr..

[bib38] Gaggiano C. (2021). Anakinra and canakinumab for patients with R92Q-associated autoinflammatory syndrome: a multicenter observational study from the AIDA Network. Ther Adv Musculoskelet Dis.

[bib39] Garlanda C., Dinarello C.A., Mantovani A. (2013). The interleukin-1 family: back to the future. Immunity.

[bib40] Gengatharan A. (2021). Adult neural stem cell activation in mice is regulated by the day/night cycle and intracellular calcium dynamics. Cell.

[bib41] Goshen I. (2007). A dual role for interleukin-1 in hippocampal-dependent memory processes. Psychoneuroendocrinology.

[bib42] Goshen I. (2008). Brain interleukin-1 mediates chronic stress-induced depression in mice via adrenocortical activation and hippocampal neurogenesis suppression. Mol. Psychiatr..

[bib43] Goshen I., Yirmiya R. (2009). Interleukin-1 (IL-1): a central regulator of stress responses. Front. Neuroendocrinol..

[bib44] Green H.F. (2012). A role for interleukin-1β in determining the lineage fate of embryonic rat hippocampal neural precursor cells. Mol. Cell. Neurosci..

[bib45] Griffin W.S. (1989). Brain interleukin 1 and S-100 immunoreactivity are elevated in Down syndrome and Alzheimer disease. Proc. Natl. Acad. Sci. U. S. A..

[bib46] Griffin W.S. (2006). Interleukin-1 mediates Alzheimer and Lewy body pathologies. J. Neuroinflammation.

[bib47] Gulino R. (2023). Hippocampal noradrenaline is a positive regulator of spatial working memory and neurogenesis in the rat. Int. J. Mol. Sci..

[bib48] Guo Q. (2024). Long-term cognitive effects of electroconvulsive therapy in major depressive disorder: a systematic review and meta-analysis. Psychiatry Res..

[bib49] Helmy A. (2014). Recombinant human interleukin-1 receptor antagonist in severe traumatic brain injury: a phase II randomized control trial. J. Cerebr. Blood Flow Metabol..

[bib50] Hovorka M., Ewing D., Middlemas D.S. (2022). Chronic SSRI treatment, but not norepinephrine reuptake inhibitor treatment, increases neurogenesis in juvenile rats. Int. J. Mol. Sci..

[bib51] Imayoshi I. (2008). Roles of continuous neurogenesis in the structural and functional integrity of the adult forebrain. Nat. Neurosci..

[bib52] Ishikawa R. (2019). Improvement of PTSD-like behavior by the forgetting effect of hippocampal neurogenesis enhancer memantine in a social defeat stress paradigm. Mol. Brain.

[bib53] Jeon S.W., Kim Y.K. (2018). The role of neuroinflammation and neurovascular dysfunction in major depressive disorder. J. Inflamm. Res..

[bib54] Jin W.N. (2021). Neuroblast senescence in the aged brain augments natural killer cell cytotoxicity leading to impaired neurogenesis and cognition. Nat. Neurosci..

[bib55] Jones M.E. (2018). Hippocampal interleukin-1 mediates stress-enhanced fear learning: a potential role for astrocyte-derived interleukin-1β. Brain Behav. Immun..

[bib56] Kirby E.D. (2012). Basolateral amygdala regulation of adult hippocampal neurogenesis and fear-related activation of newborn neurons. Mol. Psychiatr..

[bib57] Kirby E.D. (2013). Acute stress enhances adult rat hippocampal neurogenesis and activation of newborn neurons via secreted astrocytic FGF2. Elife.

[bib58] Kitazawa M. (2011). Blocking IL-1 signaling rescues cognition, attenuates tau pathology, and restores neuronal β-catenin pathway function in an Alzheimer's disease model. J. Immunol..

[bib59] Koo J.W., Duman R.S. (2008). IL-1β is an essential mediator of the antineurogenic and anhedonic effects of stress. Proc. Natl. Acad. Sci..

[bib60] Koo J.W., Duman R.S. (2009). Evidence for IL-1 receptor blockade as a therapeutic strategy for the treatment of depression. Curr. Opin. Invest. Drugs.

[bib61] Krueger J.M. (1998). Sleep. A physiologic role for IL-1 beta and TNF-alpha. Ann. N. Y. Acad. Sci..

[bib62] Küffer A. (2019). Altered overnight levels of pro-inflammatory cytokines in men and women with posttraumatic stress disorder. Psychoneuroendocrinology.

[bib63] Kuhn H., Dickinson-Anson H., Gage F. (1996). Neurogenesis in the dentate gyrus of the adult rat: age-related decrease of neuronal progenitor proliferation. J. Neurosci..

[bib64] LaSarge C.L. (2016). Disrupted hippocampal network physiology following PTEN deletion from newborn dentate granule cells. Neurobiol. Dis..

[bib65] Lazarov O. (2010). When neurogenesis encounters aging and disease. Trends Neurosci..

[bib66] Lee D.H. (2022). Neuroinflammation in post-traumatic stress disorder. Biomedicines.

[bib67] Li W. (2020). Protective mechanism and treatment of neurogenesis in cerebral ischemia. Neurochem. Res..

[bib68] Li S. (2021). NLRP3/caspase-1/GSDMD-mediated pyroptosis exerts a crucial role in astrocyte pathological injury in mouse model of depression. JCI Insight.

[bib69] Li Y.D. (2022). Hypothalamic modulation of adult hippocampal neurogenesis in mice confers activity-dependent regulation of memory and anxiety-like behavior. Nat. Neurosci..

[bib70] Li M. (2023). IL-1ra treatment prevents chronic social defeat stress-induced depression-like behaviors and glutamatergic dysfunction via the upregulation of CREB-BDNF. J. Affect. Disord..

[bib71] Liddelow S.A. (2017). Neurotoxic reactive astrocytes are induced by activated microglia. Nature.

[bib72] Lilic D. (1990). Interleukin-1 in vivo modulates trauma-induced immunosuppression. Eur. Cytokine Netw..

[bib73] Liu X. (2019). Cell-type-specific interleukin 1 receptor 1 signaling in the brain regulates distinct neuroimmune activities. Immunity.

[bib74] Liu F. (2024). Impacts of inflammatory cytokines on depression: a cohort study. BMC Psychiatry.

[bib75] Lopez-Castejon G., Brough D. (2011). Understanding the mechanism of IL-1β secretion. Cytokine Growth Factor Rev..

[bib76] Lopez-Rodriguez A.B. (2021). Acute systemic inflammation exacerbates neuroinflammation in Alzheimer's disease: IL-1β drives amplified responses in primed astrocytes and neuronal network dysfunction. Alzheimers Dement.

[bib77] Mahar I. (2014). Stress, serotonin, and hippocampal neurogenesis in relation to depression and antidepressant effects. Neurosci. Biobehav. Rev..

[bib78] Martin S.J. (2016). Cell death and inflammation: the case for IL-1 family cytokines as the canonical DAMPs of the immune system. FEBS J..

[bib79] Martin M.U., Wesche H. (2002). Summary and comparison of the signaling mechanisms of the Toll/interleukin-1 receptor family. Biochim. Biophys. Acta.

[bib80] McAfoose J., Baune B.T. (2009). Evidence for a cytokine model of cognitive function. Neurosci. Biobehav. Rev..

[bib81] McCloskey D.P. (2006). Stereological methods reveal the robust size and stability of ectopic hilar granule cells after pilocarpine‐induced status epilepticus in the adult rat. Eur. J. Neurosci..

[bib82] McEwen B.S. (1998). Protective and damaging effects of stress mediators. N. Engl. J. Med..

[bib83] Miller J.A. (2013). Conserved molecular signatures of neurogenesis in the hippocampal subgranular zone of rodents and primates. Development.

[bib84] Ming G.L., Song H. (2005). Adult neurogenesis in the mammalian central nervous system. Annu. Rev. Neurosci..

[bib85] Ming G.-L., Song H. (2011). Adult neurogenesis in the mammalian brain: significant answers and significant questions. Neuron.

[bib86] Mishra R. (2022). Augmenting neurogenesis rescues memory impairments in Alzheimer's disease by restoring the memory-storing neurons. J. Exp. Med..

[bib87] Moreno-Jiménez E.P. (2019). Adult hippocampal neurogenesis is abundant in neurologically healthy subjects and drops sharply in patients with Alzheimer's disease. Nat Med.

[bib88] Murray C.L. (2013). Endogenous IL-1 in cognitive function and anxiety: a study in IL-1RI-/- mice. PLoS One.

[bib89] Myung J. (2018). The choroid plexus is an important circadian clock component. Nat. Commun..

[bib90] Nemeth D.P. (2024). Localization of brain neuronal IL-1R1 reveals specific neural circuitries responsive to immune signaling. J. Neuroinflammation.

[bib91] Nilsson M. (1999). Enriched environment increases neurogenesis in the adult rat dentate gyrus and improves spatial memory. J. Neurobiol..

[bib92] Nutt D.J. (2008). Relationship of neurotransmitters to the symptoms of major depressive disorder. J. Clin. Psychiatry.

[bib93] Okamoto M. (2021). High-intensity intermittent training enhances spatial memory and hippocampal neurogenesis associated with BDNF signaling in rats. Cerebr. Cortex.

[bib94] Opp M.R., Obál F., Krueger J.M. (1991). Interleukin 1 alters rat sleep: temporal and dose-related effects. Am. J. Physiol..

[bib95] Overstreet Wadiche L. (2005). GABAergic signaling to newborn neurons in dentate gyrus. J. Neurophysiol..

[bib96] Overstreet-Wadiche L.S. (2006). Seizures accelerate functional integration of adult-generated granule cells. J. Neurosci..

[bib97] Park H.R. (2023). Pro-neurogenic effects of Lilii Bulbus on hippocampal neurogenesis and memory. Biomed. Pharmacother..

[bib98] Pellarin I. (2025). Cyclin-dependent protein kinases and cell cycle regulation in biology and disease. Signal Transduct. Targeted Ther..

[bib99] Preskorn S.H. (1997). Clinically relevant pharmacology of selective serotonin reuptake inhibitors. An overview with emphasis on pharmacokinetics and effects on oxidative drug metabolism. Clin. Pharmacokinet..

[bib100] Quan N. (1996). Detection of interleukin-1 bioactivity in various brain regions of normal healthy rats. Neuroimmunomodulation.

[bib101] Quan N., Whiteside M., Herkenham M. (1998). Time course and localization patterns of interleukin-1beta messenger RNA expression in brain and pituitary after peripheral administration of lipopolysaccharide. Neuroscience.

[bib102] Quan N. (1999). Induction of pro-inflammatory cytokine mRNAs in the brain after peripheral injection of subseptic doses of lipopolysaccharide in the rat. J. Neuroimmunol..

[bib103] Quintela T. (2013). Analysis of the effects of sex hormone background on the rat choroid plexus transcriptome by cDNA microarrays. PLoS One.

[bib104] Rahmani A. (2013). Neurogenesis and increase in differentiated neural cell survival via phosphorylation of Akt1 after fluoxetine treatment of stem cells. BioMed Res. Int..

[bib105] Redell J.B. (2020). Traumatic brain injury and hippocampal neurogenesis: functional implications. Exp. Neurol..

[bib106] Reynolds B.A., Weiss S. (1992). Generation of neurons and astrocytes from isolated cells of the adult mammalian central nervous system. Science.

[bib107] Roerink M.E. (2017). Interleukin-1 as a mediator of fatigue in disease: a narrative review. J. Neuroinflammation.

[bib108] Roos L.G. (2019). Higher trait reappraisal predicts stronger HPA axis habituation to repeated stress. Psychoneuroendocrinology.

[bib109] Rothwell N. (2003). Interleukin-1 and neuronal injury: mechanisms, modification, and therapeutic potential. Brain Behav. Immun..

[bib110] Ryan S.M. (2013). Negative regulation of TLX by IL-1β correlates with an inhibition of adult hippocampal neural precursor cell proliferation. Brain Behav. Immun..

[bib111] Saaltink D.J., Vreugdenhil E. (2014). Stress, glucocorticoid receptors, and adult neurogenesis: a balance between excitation and inhibition?. Cell. Mol. Life Sci..

[bib112] Saheb M. (2023). Effect of a combined program of running exercise and environmental enrichment on memory function and neurogenesis markers in amyloid-beta-induced Alzheimer-like model. Iran J Basic Med Sci.

[bib113] Samatra D., Pratiwi N.M.D., Widyadharma I.P.E. (2018). High Il-1β serum as a predictor of decreased cognitive function in mild traumatic brain injury patients. Open Access Maced J Med Sci.

[bib114] Sanz-Cabanillas J.L. (2023). Efficacy and safety of anakinra and canakinumab in PSTPIP1-associated inflammatory diseases: a comprehensive scoping review. Front. Immunol..

[bib115] Schädlich I.S. (2022). Interleukin-1 mediates ischemic brain injury via induction of IL-17A in γδ T cells and CXCL1 in astrocytes. NeuroMolecular Med..

[bib116] Schneider H. (1998). A neuromodulatory role of interleukin-1beta in the hippocampus. Proc. Natl. Acad. Sci. U. S. A..

[bib117] Semënov M.V. (2019). Adult hippocampal neurogenesis is a developmental process involved in cognitive development. Front. Neurosci..

[bib118] Shaftel S.S. (2007). Sustained hippocampal IL-1 beta overexpression mediates chronic neuroinflammation and ameliorates Alzheimer plaque pathology. J. Clin. Investig..

[bib119] Shaftel S.S., Griffin W.S.T., O'Banion M.K. (2008). The role of interleukin-1 in neuroinflammation and Alzheimer disease: an evolving perspective. J. Neuroinflammation.

[bib120] Shim H.S. (2019). Role of astrocytic GABAergic system on inflammatory cytokine-induced anxiety-like behavior. Neuropharmacology.

[bib121] Singh C. (2012). Allopregnanolone restores hippocampal-dependent learning and memory and neural progenitor survival in aging 3xTgAD and nonTg mice. Neurobiol. Aging.

[bib122] Snyder J.S. (2005). A role for adult neurogenesis in spatial long-term memory. Neuroscience.

[bib123] Snyder J.S. (2011). Adult hippocampal neurogenesis buffers stress responses and depressive behaviour. Nature.

[bib124] Sokolova A. (2009). Monocyte chemoattractant protein-1 plays a dominant role in the chronic inflammation observed in Alzheimer's disease. Brain Pathol..

[bib125] Soung A.L. (2022). IL-1 reprogramming of adult neural stem cells limits neurocognitive recovery after viral encephalitis by maintaining a proinflammatory state. Brain Behav. Immun..

[bib126] Spivak B. (1997). Elevated levels of serum interleukin-1 beta in combat-related posttraumatic stress disorder. Biol. Psychiatry.

[bib127] Spulber S. (2008). Blunted neurogenesis and gliosis due to transgenic overexpression of human soluble IL-1ra in the mouse. Eur. J. Neurosci..

[bib128] Stojakovic A. (2017). Role of the IL-1 pathway in dopaminergic neurodegeneration and decreased voluntary movement. Mol. Neurobiol..

[bib129] Sun L. (2020). Akt dependent adult hippocampal neurogenesis regulates the behavioral improvement of treadmill running to mice model of post-traumatic stress disorder. Behav. Brain Res..

[bib130] Sun L. (2021). Catalpol enhanced physical exercise-mediated brain functional improvement in post-traumatic stress disorder model via promoting adult hippocampal neurogenesis. Aging (Albany NY).

[bib131] Sung P.-S. (2020). Neuroinflammation and neurogenesis in Alzheimer's disease and potential therapeutic approaches. Int. J. Mol. Sci..

[bib132] Szczytkowski-Thomson J.L., Lebonville C.L., Lysle D.T. (2013). Morphine prevents the development of stress-enhanced fear learning. Pharmacol. Biochem. Behav..

[bib133] Tartt A.N. (2022). Dysregulation of adult hippocampal neuroplasticity in major depression: pathogenesis and therapeutic implications. Mol. Psychiatr..

[bib134] Tehranian R. (2002). Improved recovery and delayed cytokine induction after closed head injury in mice with central overexpression of the secreted isoform of the interleukin-1 receptor antagonist. J. Neurotrauma.

[bib135] Thakur A. (2022). A review on post-traumatic stress disorder (PTSD): symptoms, therapies and recent case studies. Curr. Mol. Pharmacol..

[bib136] Toda T. (2019). The role of adult hippocampal neurogenesis in brain health and disease. Mol. Psychiatr..

[bib137] Tozuka Y. (2005). GABAergic excitation promotes neuronal differentiation in adult hippocampal progenitor cells. Neuron.

[bib138] Tunc-Ozcan E. (2019). Activating newborn neurons suppresses depression and anxiety-like behaviors. Nat. Commun..

[bib139] Tuncdemir S.N. (2023). Adult-born granule cells facilitate remapping of spatial and non-spatial representations in the dentate gyrus. Neuron.

[bib140] Uddin M. (2010). Epigenetic and immune function profiles associated with posttraumatic stress disorder. Proc. Natl. Acad. Sci..

[bib141] Urbán N., Guillemot F. (2014). Neurogenesis in the embryonic and adult brain: same regulators, different roles. Front. Cell. Neurosci..

[bib142] Van Praag H., Kempermann G., Gage F.H. (1999). Running increases cell proliferation and neurogenesis in the adult mouse dentate gyrus. Nat. Neurosci..

[bib143] Videbech P., Ravnkilde B. (2004). Hippocampal volume and depression: a meta-analysis of MRI studies. Am. J. Psychiatr..

[bib144] Vivar C. (2015). Adult hippocampal neurogenesis, aging and neurodegenerative diseases: possible strategies to prevent cognitive impairment. Curr. Top. Med. Chem..

[bib145] Vivar C., Potter M.C., van Praag H. (2013). All about running: synaptic plasticity, growth factors and adult hippocampal neurogenesis. Curr Top Behav Neurosci.

[bib146] Vivar C. (2023). Running throughout middle-age keeps old adult-born neurons wired. eNeuro.

[bib147] Waheed A. (2018). A systematic review of interleukin-1β in post-traumatic stress disorder: evidence from human and animal studies. J. Interferon Cytokine Res..

[bib148] Walgrave H. (2021). Restoring miR-132 expression rescues adult hippocampal neurogenesis and memory deficits in Alzheimer's disease. Cell Stem Cell.

[bib149] Wang K.C. (2011). Interleukin-1 receptor antagonist ameliorates neonatal lipopolysaccharide-induced long-lasting hyperalgesia in the adult rats. Toxicology.

[bib150] Wang X. (2016). Traumatic brain injury severity affects neurogenesis in adult mouse Hippocampus. J. Neurotrauma.

[bib151] Wang W. (2019). Characteristics of pro- and anti-inflammatory cytokines alteration in PTSD patients exposed to a deadly earthquake. J. Affect. Disord..

[bib152] Wang J. (2025). Repetitive traumatic brain injury-induced complement C1-related inflammation impairs long-term hippocampal neurogenesis. Neural Regen Res.

[bib153] Weber A., Wasiliew P., Kracht M. (2010). Interleukin-1 (IL-1) pathway. Sci. Signal..

[bib154] Webster K.M. (2017). Inflammation in epileptogenesis after traumatic brain injury. J. Neuroinflammation.

[bib155] Wu M.D. (2012). Adult murine hippocampal neurogenesis is inhibited by sustained IL-1β and not rescued by voluntary running. Brain Behav. Immun..

[bib156] Wu A., Zhang J. (2023). Neuroinflammation, memory, and depression: new approaches to hippocampal neurogenesis. J. Neuroinflammation.

[bib157] Xia C.Y. (2023). The NLRP3 inflammasome in depression: potential mechanisms and therapies. Pharmacol. Res..

[bib158] Yagi S. (2022). Sex differences in contextual pattern separation, neurogenesis, and functional connectivity within the limbic system. Biol. Sex Differ..

[bib159] Yan X., Jiang E., Weng H.R. (2015). Activation of toll like receptor 4 attenuates GABA synthesis and postsynaptic GABA receptor activities in the spinal dorsal horn via releasing interleukin-1 beta. J. Neuroinflammation.

[bib160] Yau S.Y., Li A., So K.F. (2015). Involvement of adult hippocampal neurogenesis in learning and forgetting. Neural Plast..

[bib161] Yirmiya R., Winocur G., Goshen I. (2002). Brain interleukin-1 is involved in spatial memory and passive avoidance conditioning. Neurobiol. Learn. Mem..

[bib162] Zhang Z. (2022). Ubiquitin-specific protease 22 promotes neural stem cells stemness maintenance and adult hippocampal neurogenesis, contributing to cognitive recovery following traumatic brain injury. Neuroscience.

[bib163] Zhang K. (2023). Hyperactive neuronal autophagy depletes BDNF and impairs adult hippocampal neurogenesis in a corticosterone-induced mouse model of depression. Theranostics.

[bib164] Zhang J. (2024). Recent advances in Alzheimer's disease: mechanisms, clinical trials and new drug development strategies. Signal Transduct. Targeted Ther..

